# Introduction and Differential Diagnosis of Monkeypox in Argentina, 2022

**DOI:** 10.3201/eid2810.221075

**Published:** 2022-10

**Authors:** Adrian Lewis, Alejandro Josiowicz, Stella Maris Hirmas Riade, Monica Tous, Gustavo Palacios, Daniel M. Cisterna

**Affiliations:** Instituto Nacional de Enfermedades Infecciosas, ANLIS Dr. Carlos G. Malbran, Buenos Aires, Argentina (A. Lewis, A. Josiowicz, S.M.H. Riade, M. Tous, D.M. Cisterna);; Icahn School of Medicine at Mount Sinai, New York, New York, USA (G.P.)

**Keywords:** monkeypox, atypical hand-foot-mouth syndrome, enterovirus coxsackie, A6, viruses, sexually transmitted infections, Argentina, Bolivia

## Abstract

We report detection of cases of monkeypox virus infection in Argentina in the context of a marked increase in confounding cases of atypical hand-foot-and-mouth syndrome caused by enterovirus coxsackie A6. We recommend performing an accurate differential virological diagnosis for exanthematous disease in suspected monkeypox cases.

Global surveillance of monkeypox cases has resulted in the detection of an increasing number of suspected cases in countries to which the disease is not endemic (*1*). We report the results of a virological investigation of 9 suspected cases of monkeypox from Argentina (n = 6) and Bolivia (n = 3) detected during May 22–June 8, 2022. The investigation was conducted using World Health Organization case definitions (*2*).

We attempted laboratory diagnosis for all 9 cases by using classical and molecular methods such as electron microscopy (EM) and conventional orthopoxvirus PCR. We analyzed swab samples collected from the skin, genital lesions, or both for monkeypox screening. We performed negative staining electron microscopy using direct absorption for 10 minutes of a 10-μL sample volume on fomvar-coated 400 mesh grids. We performed staining with 1% phosphotungstic acid (*3*) and examined samples using a Zeiss EM-109 transmission electron microscope.

We extracted viral nucleic acid by using the High Pure Viral RNA kit (Roche Molecular Biochemicals, https://www.roche.com) according to the manufacturer’s instructions. We performed end-point PCR amplification by using primers EACP1 and EACP2 targeting the complete viral hemagglutinin gene, as done previously (*4*). We sequenced amplicon PCR fragments by using BigDye Terminator version 3.1 reagent in an ABI3500 Genetic Analyzer automatic sequencer (both ThermoFisher Scientific, https://www.thermofisher.com). We performed phylogenetic analysis by using the maximum-likelihood method and Tamuka 3-parameter model according to Modeltest using MEGA software (https://www.megasoftware.net). We produced bootstraps using 500 replicates. For differential diagnosis, we analyzed negative monkeypox virus (MPXV) samples by molecular methods for the detection of herpes simplex virus, varicella zoster virus, and enterovirus. We performed molecular typing of enteroviruses as previously reported (*5*).

The images obtained by EM in cases 1–3, all from Argentina, showed the presence of viral particles compatible with a member of the genus *Orthopoxvirus* (Appendix Figure 1). The phylogenetic analysis of the complete hemagglutinin genes for these viruses confirmed the identification of MPXV (West African clade) (Appendix Figure 2). Enterovirus was identified by PCR in 4 (66.7%) of the remaining 6 cases (2 from Argentina and 2 from Bolivia). Coxsackievirus A6 (CV-A6) was identified in 3 of these 4 cases. CV-A6 is usually associated with atypical hand-foot-mouth syndrome. Finally, the 6 samples analyzed were negative for herpes simplex virus and varicella zoster virus. In summary, of the 9 cases from South America with exanthematic disease that fit the definition of suspected cases, 3 (33%) cases were confirmed for MPXV and 4 (44%) cases were differentially diagnosed as CV-A6 infections.

We evaluated the clinical manifestations of all 9 cases. In the 3 laboratory-confirmed cases of MPXV, clinical manifestations included pustular lesions of heterogeneous distribution in the body, multiple painful intergluteal and perianal lesions, and genital ulcers (Table). All 3 patients reported multiple sexual partners during the previous few weeks, 2 during international travel to Spain and 1 with international travelers from countries reporting cases. No patients experienced lymphadenopathy. Patients 1 and 3 were hospitalized briefly for pain management related to their symptoms.

**Table:** Characteristics of suspected cases of monkeypox in Argentina and Bolivia*

Patient no.	Age, y/sex	Clinical manifestations	Country	Travel history	Hospital admission	Background	MPXV PCR	EV PCR/EV type	HSV PCR	VZV PCR
1	40/M	Pustular lesions on the left shoulder, sternal, cervical, right scapula, left lower limb, multiple painful intergluteal and perianal lesions	Argentina	Spain	Yes	Multiple sexual partners, HIV+	+	ND	ND	ND
2	40/M	Genital ulcer	Argentina	Spain	No	Multiple sexual partners	+	ND	ND	ND
3	36/M	Fever, headache, myalgia, back pain, maculopapular lesions and pustules	Argentina	No reported travel	Yes	Multiple sexual partners	+	ND	ND	ND
4	36/M	Vesicular lesions on the palms, soles, and perineum	Argentina	Dominican Republic	No	3-year-old son with blistering lesion on the perineum	–	–	–	–
5	43/M	Fever, maculopapular lesions and pustules	Argentina	Paraguay	No	No data	–	+/CV-A6	–	–
6	39/F	Vesicular lesions on hand, mouth, and groin area	Argentina	Dominican Republic and Colombia	No	No data	–	+/CV-A6	–	–
7	53/F	Exanthematous lesions of unspecified distribution, lymphadenopathy	Bolivia	No reported travel	No	No data	–	–	–	–
8	22/F	Exanthematous lesions of unspecified distribution, lymphadenopathy	Bolivia	Spain	No	No data	–	+/CV-A6	–	–
9	27/M	Exanthematous lesions of unspecified distribution, lymphadenopathy	Bolivia	No reported travel	No	HIV+	–	+/ND	–	–

*CV-A6, coxsackievirus A6; EV, enterovirus; HSV, herpes simplex virus; MPXV, monkeypox virus; ND, not done; VZV, varicella zoster virus; +, positive; –, negative.

The remaining 6 patients who were negative for orthopoxvirus displayed vesicular lesions in various stages on the palms, soles, and genital locations. Some reported travel from the Dominican Republic, Colombia, Paraguay, or Spain.

The epidemiologic information we collected on these monkeypox cases, together with genetic analysis, confirm that they are directly related to outbreaks in several countries in Europe (*6*) and are not linked to previous introductions in the United States (*7*; C.M. Gigante et al., unpub. data, https://www.biorxiv.org/content/10.1101/2022.06.10.495526v1). Although 1 patient did not travel, he reported direct physical contact with persons who had traveled to countries with reported cases, revealing local community transmission.

Of note, South America is experiencing a marked increase in cases of atypical hand-foot-mouth syndrome caused by CV-A6 (*8*,*9*). Unlike the classic syndrome, this atypical variant also affects young adults and occurs in unusual regions of the body, including the genital areas, and could easily be confused with monkeypox. A wide case definition makes surveillance easier, but it also emphasizes the need to perform precise differential virological diagnosis for exanthematic disease in suspected cases.

In summary, we report 3 cases of monkeypox in patients in Argentina. Six additional patients in Argentina and Bolivia had monkeypox ruled out by differential diagnosis; 4 of those cases were atypical hand-foot-mouth syndrome caused by CV-A6. We recommend considering virological diagnosis of this disease in suspected cases of monkeypox. Clinicians should be aware of the possibility for misdiagnosis related to these viral infections.

Appendix Additional information about introduction and differential diagnosis of monkeypox in Argentina, 2022
