# Supplementary material for: Introduction and Differential Diagnosis of Monkeypox in Argentina, 2022
Source: Emerg Infect Dis. 2022 Oct;28(10):2123–5. doi: 10.3201/eid2810.221075 (PMC9514367; doi:10.3201/eid2810.221075)
Supplement: Appendix — Additional information about introduction and differential diagnosis of monkeypox in Argentina, 2022 [file 22-1075-Techapp-s1.pdf]

Article DOI: https://doi.org/10.3201/eid2810.221075

Introduction and Differential Diagnosis of Monkeypox in Argentina, 2022

Appendix

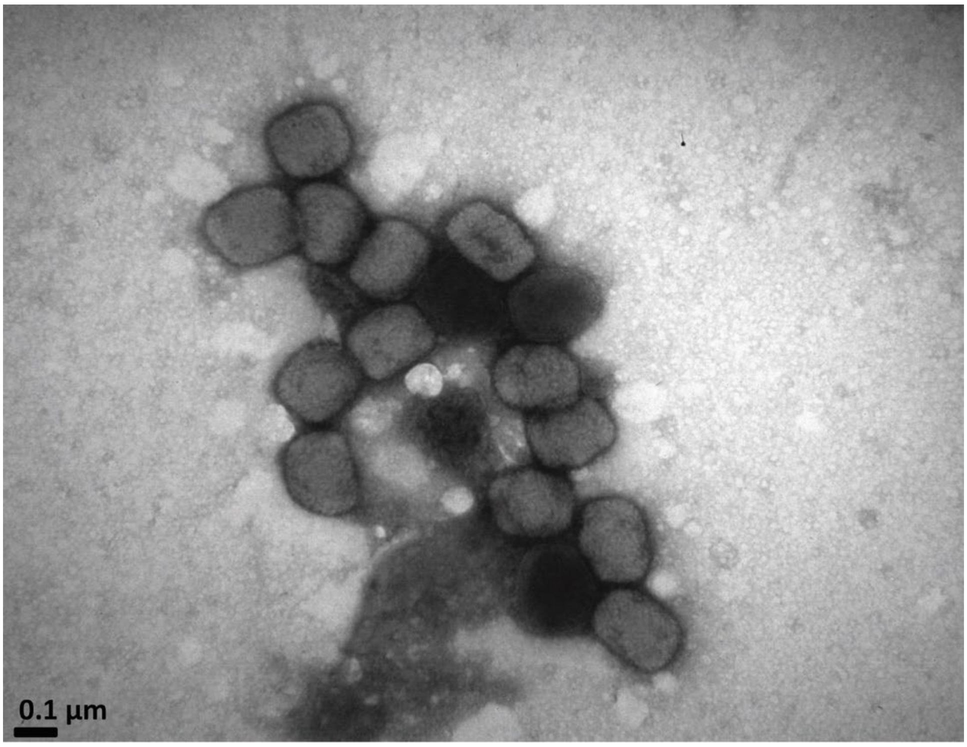

Appendix Figure 1. Electron microimage of monkeypox cases, Argentina, 2022. The virus was identified from the biologic fluid of the vesicular lesions of a patient with a presumptive clinical diagnosis of monkeypox.

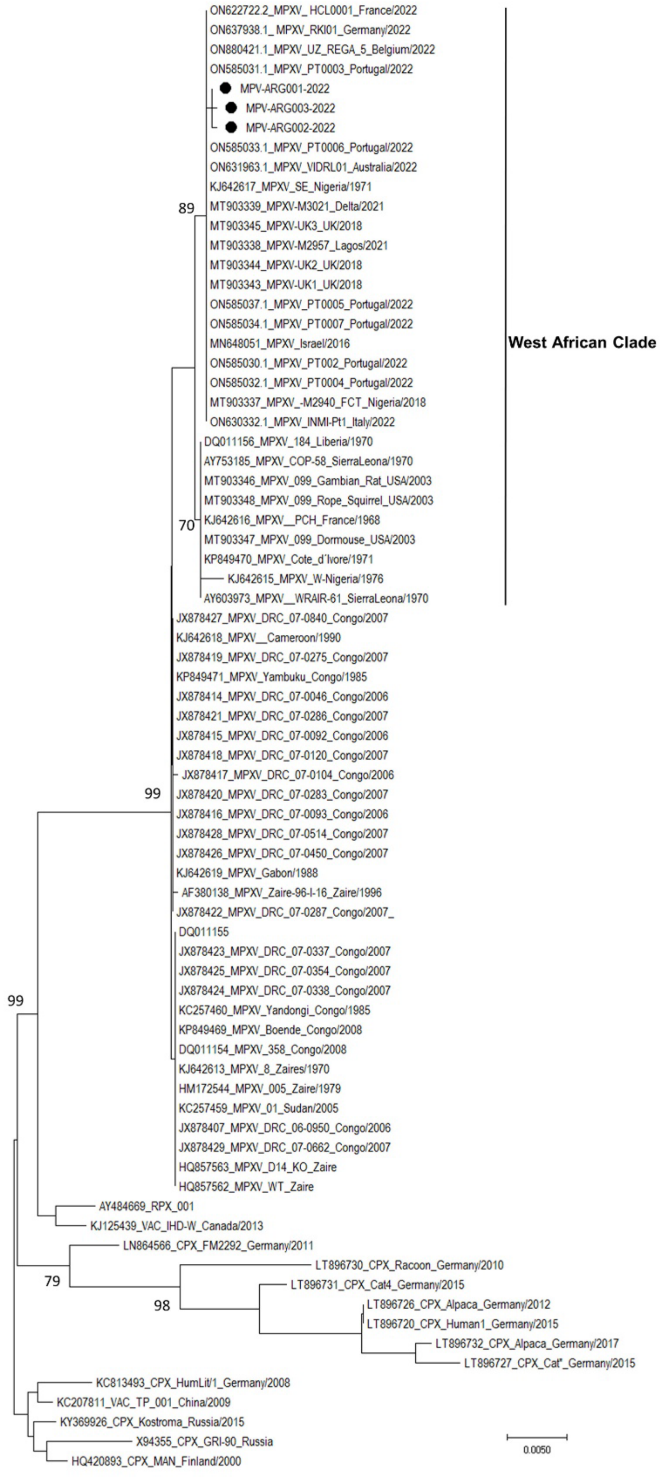

Appendix Figure 2. Phylogenetic analysis of 3 monkeypox cases, Argentina, 2022. The 3 Argentine samples are highlighted with a black circle (ON800897.1, ON720962.1, and ON669283.1). Sequences from other countries where an increase in cases of monkeypox virus has occurred are included. The following information is included in the tree: Genbank accession number/virus/strain name/country/year. CPX, cowpox virus; MPXV, monkeypox virus; RPX, rabbitpox virus; VAC, Vaccinia virus. Bootstrap results are included and only those with bootstrap values >70% are shown.
